# The forest, the trees, and the leaves across adulthood: Age-related changes on a visual search task containing three-level hierarchical stimuli

**DOI:** 10.3758/s13414-021-02438-3

**Published:** 2022-01-10

**Authors:** Sabrina Bouhassoun, Nicolas Poirel, Noah Hamlin, Gaelle E. Doucet

**Affiliations:** 1 Université de Paris, LaPsyDÉ, CNRS, F-75005 Paris, France; 2 GIP Cyceron, Caen, France; 3 Institut Universitaire de France (IUF), Paris, France; 4 Institute for Human Neuroscience, Boys Town National Research Hospital, 14090 Mother Teresa Lane, Boys Town, Omaha, NE 68010 USA

**Keywords:** Visual processing, Hierarchical stimuli, Healthy aging, Local versus global

## Abstract

Selecting relevant visual information in complex scenes by processing either global information or local parts helps us act efficiently within our environment and achieve goals. A global advantage (faster global than local processing) and global interference (global processing interferes with local processing) comprise an evidentiary global precedence phenomenon in early adulthood. However, the impact of healthy aging on this phenomenon remains unclear. As such, we collected behavioral data during a visual search task, including three-levels hierarchical stimuli (i.e., global, intermediate, and local levels) with several hierarchical distractors, in 50 healthy adults (26 younger (mean age: 26 years) and 24 older (mean age: 62 years)). Results revealed that processing information presented at the global and intermediate levels was independent of age. Conversely, older adults were slower for local processing compared to the younger adults, suggesting lower efficiency to deal with visual distractors during detail-oriented visual search. Although healthy older adults continued exhibiting a global precedence phenomenon, they were disproportionately less efficient during local aspects of information processing, especially when multiple visual information was displayed. Our results could have important implications for many life situations by suggesting that visual information processing is impacted by healthy aging, even with similar visual stimuli objectively presented.

## Introduction

In everyday life, we are continuously confronted with complex visual scenes. To act efficiently within our environment and achieve goals, we must select relevant visual information in those complex scenes and process either global information (e.g., a forest) or local parts (e.g., trees). The general challenge in studying global and local processes comes from the fact that in a real-world situation, the global level (e.g., the whole/the forest) can be predicted from the identity of the local level (e.g., the features/the trees) and vice versa. An essential condition for addressing this issue was the development of experimental materials that included global and local levels of information that could be obtained independently. A traditional and elegant method consists of using hierarchical stimuli with global large forms composed of arrangements consisting of small local elements (Navon, 1977). Paradigms utilizing hierarchical stimuli in adults revealed a global precedence phenomenon that indicates a global advantage (i.e., global processing is faster than local processing) and a global interference (global processing interferes with the processing of local features, e.g. Poirel et al., 2008). Moreover, it has also been shown that when both local and global information are in conflict, global information must be inhibited to process local information, but the reverse is not true (Poirel et al., 2014). Thus, since the seminal Navon (1977) work (see also Navon, 2003), it is now well established that adults usually perceive the “forest before the trees” (see Kimchi, 1992, for a general review regarding global precedence phenomenon). It has been suggested that the global precedence phenomenon could be linked to the neural “coarse-to-fine” model (i.e., global-to-local), in which the global information is preferentially conveyed by the low spatial frequencies through the rapid magnocellular visual channels (Hegdé, 2008). This process allows an initial global perceptual analysis of visual inputs, which then guides the subsequent analysis of local information (conveyed via the high spatial frequencies and the slow parvocellular visual channels) through feedback signals into low-level visual areas (Bullier, 2001; Hegdé, 2008; Kauffmann et al., 2014).

Given that during daily life situations many objects are present in the environment, studies have been conducted to refine the attentional priority during visual processing. Visual search paradigms were proposed to investigate how participants detected a specific target among various numbers of visual distractors (Treisman & Gelade, 1980). Results revealed that when a target only differed from the distractors with respect to one simple feature (e.g., color, shape or orientation), the time required to detect this target was not dependent on the number of distractors presented in the display since the target is quickly and easily identified (i.e., reflecting parallel, effortless, and efficient processing). Conversely, when a target differed from distractors by a combination of several features, the time required for detection increased with the number of distractors (i.e., reflecting serial, effortful, and less efficient processing). It is worth noting that effects reported in visual search tasks are consistent with the effects reported in global/local studies. In a global/local feature visual search task, Kimchi et al. (2005) proposed a design in which the target differs from distractors by only one feature either at the global or local level. The authors showed that when the global form contained many small elements, global processing was effortless and efficient (i.e., response times (RTs) did not vary according to the number of distractors on the display), whereas local processing was effortful and less efficient (i.e., RTs increased with the number of distractors present on the display). These results were replicated by Krakowski et al. (2015, experiment 3), suggesting that when multiple hierarchical stimuli are present on a display, they are processed from global to local analysis, with an effortless processing for global information and an effortful processing for the local ones.

Over the last century, the effects of aging on the processing of global/local hierarchical stimuli have been carried out by several studies. Indeed, numerous studies argued that global/local processing could be linked to the efficiency of a wide range of subsequent processing such as reading, memory processing or social cognition across the lifespan (e.g., Gerlach & Starrfelt, 2018; Insch et al., 2012; Oken et al., 1999; Zappullo et al., 2020). Particularly, age-related changes in global/local processing could impact critical cognitive processes such as face recognition and social cues processing (e.g., Insch et al., 2012; Oken et al., 1999). For instance, Insch et al. (2012) suggested that local bias in late adulthood may, at least in part, be predictive of poorer ability in decoding actions or emotions. In addition, numerous studies have described that individual differences in global precedence can partly predict performance during both face and object recognition (e.g., Gerlach & Starrfelt, 2018; Richler et al., 2011; Wang et al., 2012). More broadly, age-related changes in selective attention could impact subsequent processing such as episodic encoding during memory processing (Powell, et al., 2018). Taken together, these studies highlight the need to further clarify the effects of healthy aging on the allocation of attentional resources during global/local processing. In the present work, we aim to use a visual search paradigm to assess the effortfulness of global/local processes across adulthood. A visual search paradigm allows to estimate the capacity needed to process a predefined target presented among an increasing number of distractors (e.g., Treisman & Gelade, 1980). It has been suggested that such a paradigm can be used to estimate visual attention efficiency on a continuum from efficient to non-efficient search (e.g., Wolfe, Cave, & Franzel, 1989). Age-related changes regarding local/global processes could provide promising clues to better understand the age-related trajectories of subsequent cognitive processing. To our knowledge, no study to date has explored the changes in attentional demands during both global and local target visual searches in late adulthood.

According to previous studies, global processing was suggested to be affected by age with a decline of global precedence in older participants (Oken et al., 1999; Staudinger et al., 2011). Previous findings from Staudinger et al. (2011) showed, for example, that older adults were less impacted by the variation of the numbers of local elements than younger adults, suggesting a deficit in global perception. In line with these results, other research has also described age-related changes from global-to-local bias in healthy aging (Agnew et al., 2016 ; Insch et al., 2012 ; Lux et al., 2008). For instance, Lux et al. (2008) found a significant prevalence of local processing advantage during both directed and divided attention tasks in older adults compared to younger adults. Conversely, Akshoomoff et al. (1993) reported a detrimental age effect on the processing of local information, suggesting that only effortful information was affected by age. Furthermore, it has been suggested that older participants had disproportional difficulties with inhibiting global interference to efficiently process the local elements (Wiegand et al., 2015). Other studies have evidenced that even if older participants were slower than younger participants to identify hierarchical stimuli, age did not affect the global precedence phenomenon *per se* (Bruyer et al., 2003; Bruyer & Scailquin, 2000). This latter finding was in line with a Roux and Ceccaldi study (2001) that described a global advantage in both young and older adults. These previous studies were particularly focused on global advantage and global interference effects, and used different experimental designs (e.g., different stimuli type, experimental designs). Such variability in design may have led to differences in effortfulness-related results among these studies and could explain, at least in part, the inconsistency in the literature. To date, no study has investigated the impact of effortfulness variations on responses during global/local processes in younger and older participants. In agreement with Staub et al. (2015) findings regarding the effortfulness evolution with age, it seems possible that age-related changes affect both global and local levels, with global processing (which is effortless in younger adults) becomes effortful in older adults, while local processing becomes even more effortful in late adulthood.

Recently, we proposed a novel multiple-level hierarchical stimuli task to further investigate global/local visual processing by adding an intermediate level to the traditional two hierarchical levels (Krakowski et al., 2015, 2016). This addition was implemented because objects present in our environment have more than two global/local levels of processing, which further allows investigation into the dichotomy between global and local levels via a three-level hierarchical stimuli task (i.e., global forms that are composed of intermediate forms, which in turn are composed of local elements). Using three-level hierarchical stimuli, previous findings have demonstrated that the global advantage occurred on a continuum during an identification task (Rijpkema, van Aalderen, Schwarzbach, & Verstraten, 2007). Particularly, the global level was identified more quickly than the intermediate level, which was identified more quickly than the local level (Rijpkema et al., 2007). This pattern of results was replicated and was shown to only occur when there was no attentional competition from the presence of distractors, suggesting that it did not reflect the prioritization of attentional resources to hierarchical levels during visual processing in a multi-element display (Krakowski et al., 2015). During a visual search task, we identified different effects by evidencing that the local level was consistently disadvantaged in comparison to the global and intermediate levels. Importantly, while using Navon-like stimuli provides information on whether a level is processed faster compared to the other levels, it does not address whether it is related to an advantage or disadvantage of a level over another one during the competition for attentional resources. Using an intermediate level resolved this issue, by determining that the intermediate level (which is both local and global in nature) was processed the same way than the global information, whereas local information was processed less efficiently during the visual search task (Krakowski et al., 2015). This finding has provided a more in-depth analysis of the habitual opposition between global and local processing, by showing that the global information is not necessarily the only one that has an advantage in terms of effortless and efficient processes. In contrast, local information was systematically disadvantaged over both intermediate and global information when competing for the same attentional resources. In other words, this three-level visual search task has the advantage to identify whether there is a global advantage (i.e., the global level is the only level that can be efficiently processed) or a local disadvantage (i.e., the local level is the only level that is inefficiently processed). To investigate the age-related changes regarding the effortfulness of global/local processes, the present study aimed to uncover whether healthy aging impacts the efficiency of processing global, intermediate, and/or local levels during a visual search task.

In the present work, we used a three-level stimuli search task (Krakowski et al., 2015), on 50 healthy adults from 20 to 73 years of age in order to identify how attention is spread across items and how visual distractors are processed in older relative to younger adults. This task also included a range of distractors that had to be ignored to correctly perform the task. Participants were presented with a visual search task in which a predefined target (i.e., a square) was either present or not at one of the three levels of hierarchical stimuli. In order to investigate the ability to resist visual distractors, we varied the number of distractors on the screen while the target stimulus was simultaneously presented (Krakowski et al., 2015). Consistent with the global precedence phenomenon, we hypothesized that younger adults would process global targets faster than local targets. Moreover, in agreement with previous results that showed a local disadvantage, we also expected that the intermediate levels would be processed the same as global levels and faster than the local level. Regarding older participants, we hypothesized that they might process the task slower than younger adults, particularly during the most attentionally demanding local conditions. Since previous studies demonstrated that the intermediate level was processed the same as the global level, we also assumed that older participants would process intermediate levels as global levels. Finally, in agreement with the concept that an impairment to disengage from global distracting information, along with local processing that becomes even more effortful with age, we hypothesized that older participants should be significantly slower than younger participants when the number of distractors increased, particularly during the most demanding local target detection.

## Method

### Participants

We recruited a total of 50 healthy individuals and divided them into two age groups. One younger adult group of 26 participants (mean age ± sd = 25.7 ± 3.5 years, age range: 20.2-32.8 years; 14 females) and one older adult group of 24 participants (62.0 ± 4.4, age range: 55.4-73.5 years; 17 females). The age cut-off for the older group (i.e., 55 years old minimum) was based on the age criterion used in Alzheimer’s Disease Neuroimaging Initiative (ADNI) database (Jack et al., 2008; Petersen et al., 2010).

An a priori power analysis using G*Power 3.1 (Faul, Erdfelder, Lang, & Buchner, 2007) was conducted with a mixed 2x3x4 design with one between-subject factor of group (younger vs. older adults) and two within-subject factors (level of target occurrence: local vs. intermediate vs. global; number of distractors: 0 vs. 1 vs. 3 vs. 5) indicated that a sample size of 40 participants (20 per group) would be sufficient to detect a medium effect size (f = .25) with a power of .80 and an alpha of .05. All participants were free of psychiatric or neurological disorders and had normal or corrected-to-normal vision. Groups did not significantly differ in sex (*p*=.2), handedness (*p*=.06), education level (*p*=.7) or Mini-Mental State Examination (MMSE) score (Younger: mean = 29.2 ± 1.1, range: [26-30]; Older: mean = 28.5± 1.8, range: [25-30], *p*=.10). The study was approved by the Institutional Review Board for Research with Human Subjects at Boys Town National Research Hospital.

### Stimuli

**Fig. 1:**
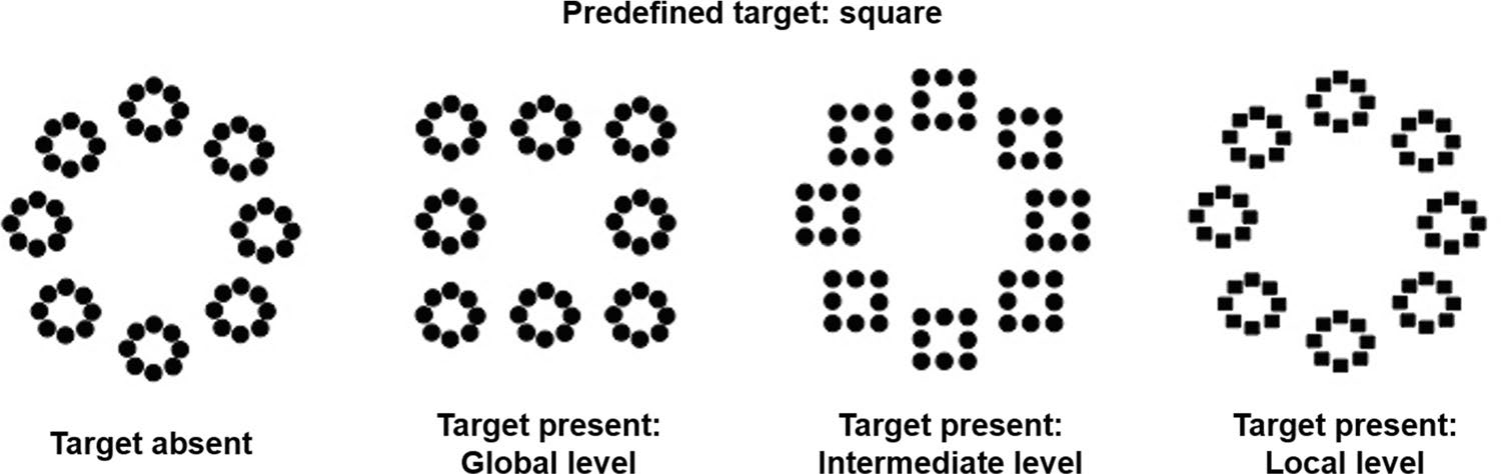
Hierarchical stimuli with three hierarchical levels. In the task used in the present experiment, a square is the predefined target and could be absent (left) or present at global, intermediate or local levels

Three-level hierarchical stimuli composed of geometrical forms (i.e., circle, square) at each level (i.e., global, intermediate, and local, Fig. 1) were used in Krakowski et al. (2015). Participants had to determine whether a square was present at any level of the hierarchical figure and respond by pressing the ‘1’ button of the keyboard to respond “square present”, and the ‘2’ button to respond “square absent”. The actual target was present in half of the trials.

**Fig. 2:**
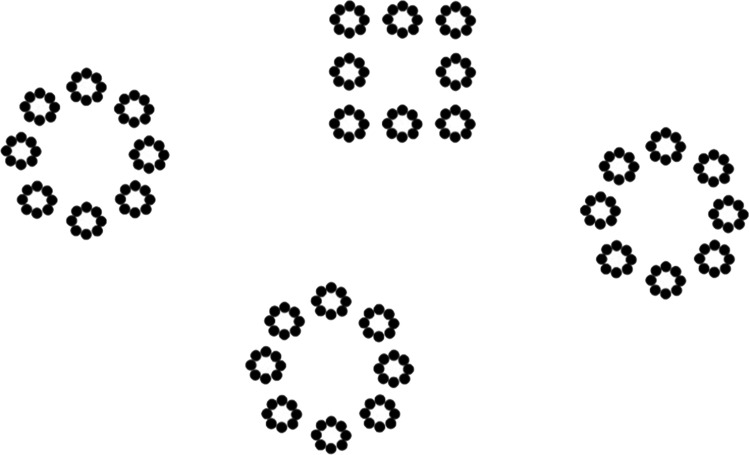
Example of present-target trials. Here, an example of a target present at the global level with three distractors is presented. It should be noted that targets could appear equally often at the global level, the intermediate level or the local level, and there could be zero, one, three or five distractors in the display

One, two, four or six three-level hierarchical stimuli were presented at the same time on the screen. In the present-target trials, only one hierarchical stimulus contained the target, which appeared at only one level (global, intermediate or local; Fig. 2). Thus, in present-target trials, there could be zero, one, three or five distractors. In the absent-target trials, there was no square target: circles were presented at all three levels for all the stimuli on the screen. These distractors were also three-level hierarchical stimuli, but with no target (i.e., all levels represent a circle).

### Procedure

The visual search task was presented using a laptop computer with a 11.6-inch screen (refresh rate: 59 Hz) running the E-Prime 3 software application (Psychology Software Tools). The participants viewed the stimuli at a distance of approximately 40 cm. Each of the local elements fits within the confines of virtual square of 0.29° in width and in height. Intermediate geometric figures were 0.86° in width and in height, and global figures were 3.72° in width and in height. Present-target items and absent-target items appeared equally often in each virtual quadrant of the screen.

Participants started with a training session consisting of 16 trials and were instructed to respond as accurately and as quickly as possible. Participants then performed two blocks of 48 trials each, including 24 present-target trials (*i.e.*, 6 trials without any distractors, 6 trials with 1 distractor, 6 trials with 3 distractors and 6 trials with 5 distractors) and 24 absent-target trials in each block (*i.e.*, 6 trials per number of hierarchical figures appearing on the screen: 1, 2, 4 or 6 hierarchical figures). The trials were randomized within blocks. In the present-target trials, the target appeared equally often at the global, intermediate, and local levels (for a total of 4 trials per block). Each trial started with the presentation of a blank screen (500 ± 250 ms), then a stimulus was displayed. The stimulus remained on the screen until the participant provided an answer. Response times (RTs) and accuracy were recorded. All participants responded with their right hand.

### Statistical analyses

The present-target trials and the absent-target trials, as well as accuracy rates and RTs, were analyzed separately. Because participants were highly accurate, we performed normality tests (i.e., Shapiro-Wilk tests) on the accuracy data separately for target-present and target-absent trials. The results of the Shapiro-Wilk tests showed that the accuracy rates did not follow a normal distribution (all ps<.001). Thus, non-parametric analyses (i.e., Kruskal Wallis tests) were conducted for the accuracy rates, while parametric analyses (see detail below) were performed on the RTs using Jamovi software (version 1.2.27).

For the present-target trials, RTs for correct responses were included in a three-factor repeated-measures analysis of variance (ANOVA) that included the age groups as a between-subject factor (younger or older adults), and both the level of target occurrence (global, intermediate or local), and the number of distractors (0, 1, 3 or 5) as within-subject factors. For the absent-target trials, RTs for correct responses were included in a two-factor repeated-measures ANOVA that included a between-subject factor of group (younger *or* older) and a within-subject factor of number of stimuli present on the display (1, 2, 4 or 6). When significant, post-hoc analyses were further conducted using multiple comparison t-tests, with Bonferroni correction. Additional planned comparisons were performed to investigate the advantage/disadvantage of one level in relation to another during the competition for attentional resources by comparing the mean differences in RTs between the level of target occurrence for each group (e.g., RTs for global target *minus* RTs for local target for younger participants compared to the related score for older participants).

Lastly, we also performed search slope analyses using mean RTs for each number of stimuli on display (i.e., set size). Such analysis allows for further investigation into the added cost of increasing number of distractors during the visual search task. RT x set size slopes were calculated using linear regressions that were computed separately for each participant (Statgraphics Centurion 19 software), with RTs for each level of target occurrence conditions (global, intermediate, local) as the dependent variable, and set size (0, 1, 3, 5, for target-present) as the independent variable. According to our hypotheses and the results from the ANOVAs on RTs, we performed within and between groups planned slopes comparisons for each level of target occurrence using paired t-tests and two-sample t-tests, as appropriate. Significant results at p<0.05 after applying a Bonferroni correction are reported.

## Results

### Accuracy data analyses

Younger and older groups were highly accurate and did not significantly differ in accuracy rates for any of the conditions (present-target: 96.7% ± 0.45 and 98.2 % ± 0.45 (mean ± sd), ps>0.23; absent-target: 99.1% ± 0.77 and 98.1 % ± 0.77 for younger and older adults, respectively, ps>0.79).

### Response times analyses of present-target trials

**Fig. 3:**
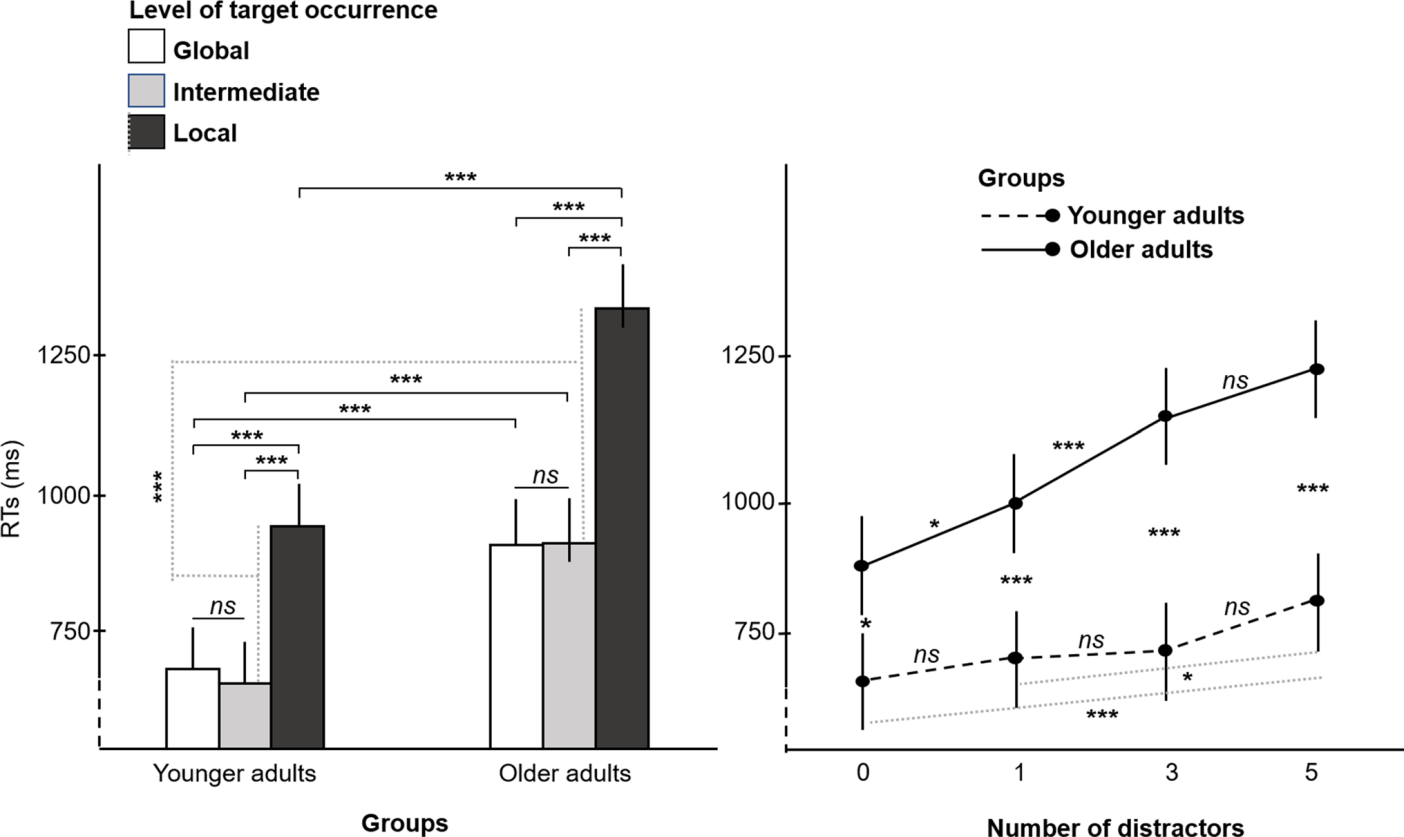
Interaction between the age group and the attentional level of the target occurrence in present-target trials (left panel) and between the age group and the number of distractors in present-target trials (right panel). *p <.05, ***p<.001, *ns* = non-significant, error bars indicate standard errors of the means. RTs: Response Times. All statistics have been corrected for multiple comparisons.

The repeated-measure ANOVA conducted on the RTs revealed a main effect of group (*F* (1,48) = 39.3, *p* <.001, η_p_^2^=.45). Overall, younger adults were faster (mean ±sd= 768 ± 230 ms) than older adults (1059 ± 404 ms). There was also a significant main effect of the level of target occurrence (*F* (2,96) = 120.86, *p* <.001, η_p_^2^=.72) and a significant group × level of target occurrence interaction (*F* (2,96) = 5.57, *p* = .005, η_p_^2^=.10). As shown in Fig. 3 (left panel), both age groups did not significantly differ in RTs between the global and intermediate levels, suggesting a global precedence effect for both conditions (global: 683 ± 39 ms and 911 ± 39 ms; intermediate: 658 ± 39 ms and 913 ± 39 ms for younger and older participants, respectively, *p*’s =1). In contrast, both groups showed slower RTs for the local level than for both global and intermediate levels (local: 945 ± 39 ms and 1335 ± 39 ms for younger and older participants, *p*’s <.001). The group × level of target occurrence interaction revealed that older participants were disproportionately slower than younger participants during local processing compared to both global (RTs for global target *minus* RTs for local target level: 263 ms and 424 ms for younger and older participants, respectively, *p* = .002) and intermediate processing (RTs for intermediate target *minus* RTs for local target level: 287 ms and 422 ms for younger and older participants, *p*=.025). Conversely, there was no significant difference between younger and older participants during intermediate processing compared to global processing (*p*=0.588).

**Fig. 4:**
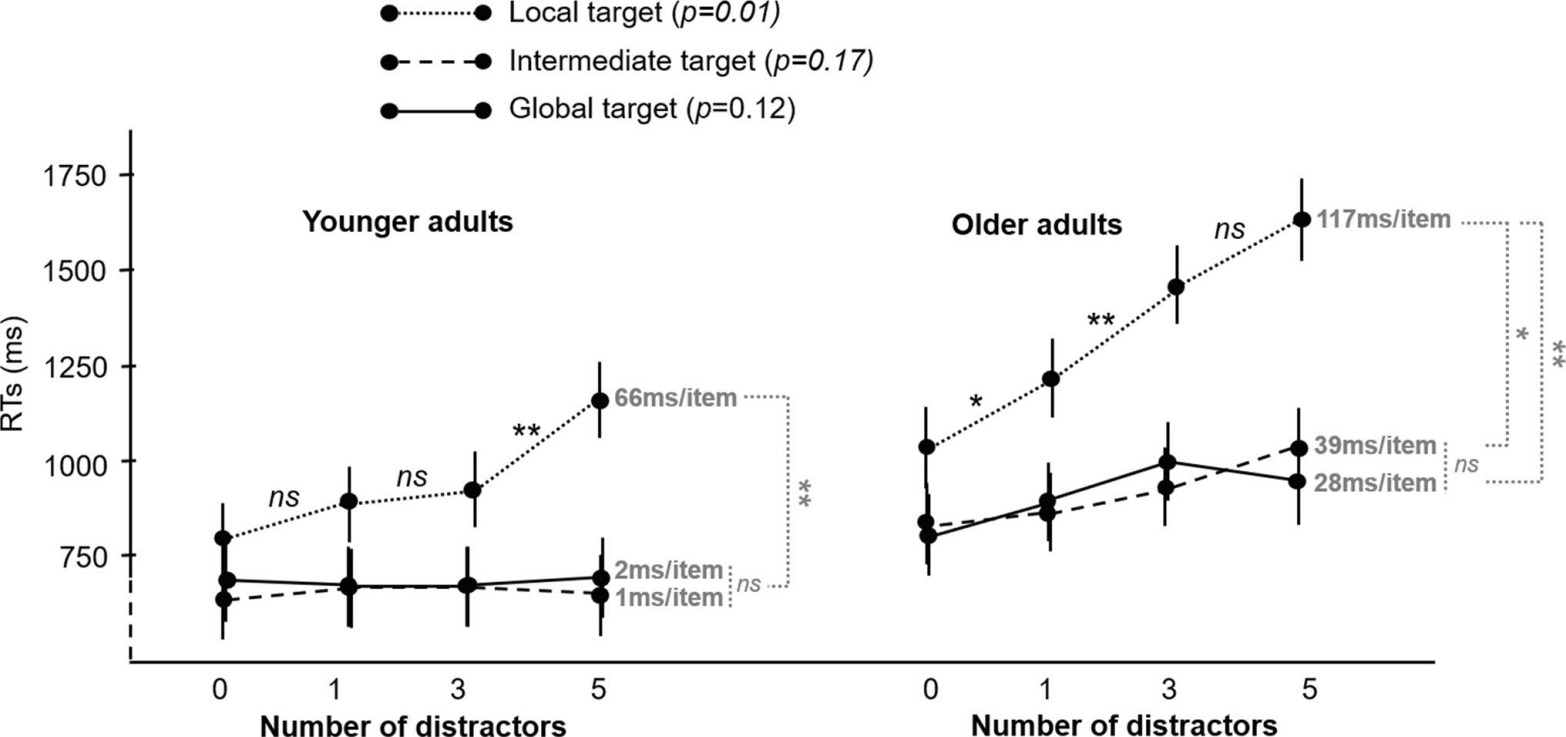
Response times by attentional level and number of distractors in present-target trials, in each age group. The RT x Set size slopes for each level are indicated in grey. *p <.05, **p <.01, *ns* = non-significant, error bars indicate standard errors of the mean. All statistics have been corrected for multiple comparisons. The *p* values mentioned on the target legend refer to group x number of distractors interactions

Furthermore, a main effect of the number of distractors (*F* (3,144) = 34.51, *p* <.001, η_p_^2^=.42) and a significant group × number of distractors interaction (*F* (3,144) = 8.35, *p* <.001, η_p_^2^=.15; Fig. 3 right panel) were also revealed. Even though all participants presented a general increase in RTs with higher number of distractors, this effect was more pronounced for older participants than for younger participants (Fig. 3, right panel). Differences between RTs for older participants differed as soon as one distractor was present on the display (*p*=.03; 1 vs. 3 distractors: *p*<.001), whereas for younger participants RTs differed more progressively with an increasing number of distractors (*p*=.001 and *p*=.05 for 0 vs. 5 and for 1 vs. 5 distractors, respectively). Finally, there was no group × level of target occurrence x number of distractors interaction (*F* (6,288) = 1.17, *p*=0.324). According to our hypotheses, we performed planned analyses regarding the impact of the number of distractors on younger and older groups for each level of target occurrence. Repeated-measures ANOVAs were thus carried out for each level of target occurrence to specifically reveal how the number of distractors impacted RTs of younger and older groups. A significant group x number of distractors interaction was only found for the local target occurrence (*p*=.012), whereas the variation of the number of distractors did not interact with the group for global (*p*=.10) and intermediate targets (*p*=.17, see Fig. 4). Thus, through increasing RTs, older participants were more impacted by the increasing number of distractors during the local visual search, but not the other two levels, compared to the younger participants.

### Search slopes analyses of present-target trials

Paired T-tests were conducted to compare the slopes between levels of target occurrence over the increasing number of distractors (i.e., local vs global, local vs intermediate, intermediate vs global) within the younger and older participants separately. These analyses revealed that there was no difference in the slopes between global and intermediate processing, in both younger (2ms /item ± 27 and 1ms/item ±19, for global and intermediate level, respectively, *p*=1) and older groups (28ms/item ± 63 and 39ms/item ±88, for global and intermediate level, respectively, *p*=1; Fig. 4). As expected, both groups exhibited greater search slopes during local processing (66ms/item ± 59 and 117ms/item ± 82, for younger and older participants, respectively) compared to the slopes measured during the other two conditions (*p*s<.025).

Two sample t-tests were conducted to compare the younger and older participants’ search slopes for each level of target occurrence. These comparisons revealed that there were no significant differences in the search slopes between the younger and older participants during global (1 ms/item ±27 and 28.3 ms/item ± 63, for younger and older participants, *p*=.106) and intermediate processing (1ms/item ±19 and 38.5ms/item ±88, for younger and older participants, *p*=.082) with increasing number of distractors. Conversely, there was a significant difference in the search slopes during the local condition between the two groups (*p*=0.028). Older participants were disproportionately negatively impacted by the increasing number of distractors and exhibited around twice as slow RTs during local processing compared to the younger participants (66ms/item ±59 and 117ms/item ± 82, for younger and older participants, respectively; Fig. 4).

### Response times analyses of absent-target trials

**Fig. 5:**
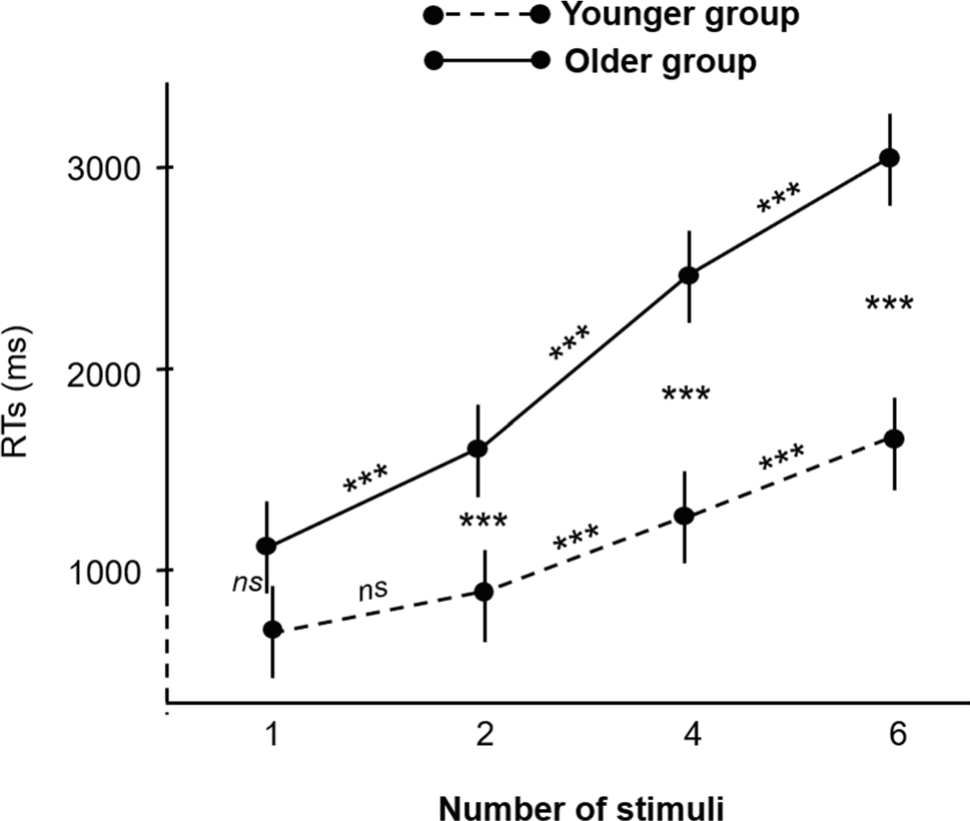
Response times by number of stimuli in absent-target trials, in each age group. ***p <.001, *ns* = non-significant, error bars indicate standard errors of the means. All statistics have been corrected for multiple comparisons.

The repeated-measures ANOVA revealed main effects of group (*F* (1,48) = 40.8, *p* <.001, η_p_^2^=.46) and number of stimuli present on the display (*F* (3,144) = 222.6, *p* <.001, η_p_^2^=.82). This analysis also revealed a significant group × number of stimuli interaction (*F* (3,144) = 24.2, *p* <.001, η_p_^2^=.34). As shown in Fig. 5, even though RTs did not differ between younger and older participants when 1 stimulus was present on the display (*p*=.09), RTs increased strongly with the number of stimuli for older as opposed to younger participants (all *p* values <.001).

## Discussion

Over the last century, psychologists have tried to understand how attentional resources are distributed in a visual display. Seminal publication evidenced the so called “global precedence phenomenon” (Navon, 1977), characterized by (1) a processing advantage of the global over the local information and (2) an interference from the global information during the processing of the local information. The current study was designed to investigate the impact of healthy aging on global precedence during a visual search task including, for the first time to our knowledge, three-levels of hierarchical stimuli. Accuracies were particularly high with ceiling effects in both younger and older participants, suggesting that the task was appropriate for the evaluation of global, intermediate, and local abilities during visual search within both age groups. In younger adults, the expected effects from previous studies were exactly replicated; the intermediate level was processed as a global level and significantly faster than the local level (Krakowski et al., 2015). Moreover, during the local condition RTs increased with increasing number of distractors present on the display, suggesting an attentional demanding condition that leads to less efficient search.

In the current study, the older participants also presented a global precedence phenomenon, as (1) they performed faster during global and intermediate levels as opposed to the local level, and (2) their RTs increased with the increasing number of distractors during local target detection, which also occurred in younger participants. Interestingly, our results showed that older participants were disproportionately less efficient during local processing; older participants processed local information more slowly than younger participants and they were impacted as soon as one distractor was present on the display. In comparison, younger adults were slower only when a minimum of five distractors were present on the display. Furthermore, RTs for the absent target condition had a stronger increase as a function of number of stimuli for the older participants than for the younger participants. Importantly, we do not believe that these results are simply due to a general slowing of processing ability in older participants because they were not slower to respond to absent target trials when one hierarchical figure was presented on the display (i.e., a single three-level hierarchical stimuli to process, no distractors) compared to the younger participants. Furthermore, neither group was influenced by the number of distractors present on the display during both global and intermediate processes, indicating a preservation of information processing with healthy aging. This suggests that: (1) global and intermediate information are processed equivalently in both age groups and (2) when multiple information is present on a display, there seems to be a reduction in attentional efficiency during local processing, though it’s still preserved as indicated by equivalent accuracy rates, in older adults compared to younger adults, with a stronger interference from the distractors information during local visual search processing (Krakowski et al., 2015). In short, regardless of the number of distractors, global and intermediate processes seem to remain efficient in middle-to-late adulthood, whereas effortful and less efficient local detail processing seems to be more sensitive to healthy aging. The specific age-related local processing changes that occur in late adulthood is consistent with previous studies that pointed out difficulties regarding the selection of local versus global stimuli (i.e., low over high saliency, Tsvetanov et al., 2013), which is associated with a reduced and delayed allocation of visuospatial attention to a target with aging (Lorenzo-López et al., 2008). In agreement with these findings, the present results suggest a decline in effortless and efficient processing with healthy aging; older adults do appear to be less able to voluntarily move their attention from one item in a visual display to another during effortful and less efficient processing (Trick & Enns, 1998). This assumption is also consistent with previous findings from tasks that involved identifying everyday life objects in older adults (Brennan et al., 2017), with the local details of a visual scene being particularly impaired later in life.

The observed greater disadvantage during local information processing in healthy older adults is also in line with results from spatial frequencies studies. It has been suggested that performance for high spatial frequencies that convey local information (Boeschoten et al., 2005) is diminished for older participants during natural scene categorization (Ramanoël et al., 2015). Consistently with the current work, low spatial frequency processing that conveys global information was not affected by age. Ramanoël et al. (2015) also evidenced that high spatial frequency processing impairment in older adults was associated with a reduced activation of a brain network including the parahippocampal place area, the right inferior occipital gyrus, and the temporo-parietal regions. These regions, particularly the temporo-occipito-parietal network, have been suggested to play a key role during global/local processing (Delis et al., 1986; Fink et al., 1996; Weissman & Woldorff, 2005) and could thus be impacted by aging. Moreover, a preservation of the “coarse-to-fine” way of processing with older age--that refers to the global precedence phenomenon in the present study--was also evidenced by Musel et al. (2012). Based on an idiosyncratic presentation of coarse-to-fine and fine-to-coarse stimuli sequences, Musel et al. (2012) suggested that even if older adults were more efficient to coarse-to-fine than to fine-to-coarse sequences, they presented an adaptation of visuo-spatial strategy regarding high frequency detail processes. This supports the present findings regarding the hypothesis of a progression of local processing of visual information throughout the lifespan.

The increasing challenge in the older group to find a local target when many distractors were present on the display could also be due to an impairment of executive function with older age. Aziz et al. (2021) evidenced a link between working memory resources and visual search abilities that differed between early and late adulthood; while younger adults showed a strong link between working memory and effortful search performance in the visuospatial domain, older adults had this association only in the verbal domain. As a matter of fact, older adults may be more inclined to rely on verbal strategy, which is a compensatory mechanism that is typically more preserved in aging than visuospatial strategy (Jenkins et al., 2000; Murre et al., 2013). In the current task, a verbal strategy is unlikely to be applicable to correctly perform the present global/local visual search task, which prevents the use of compensatory mechanisms and further could lead to increasing difficulties to deal with distractors present on the display during the local effortful condition in the older group. Moreover, an increasing challenge to deal with many distractors during local detail processing rests upon one’s inhibitory control ability, which is neurologically associated with the prefrontal cortex and seems to decline with older age (Dempster, 1992). It has been suggested that resistance to the global visual distraction during local processing is strongly linked to inhibitory control (Poirel et al., 2014). It seems conceivable that the inhibitory control involvement, which is necessary to efficiently avoid global information processing to properly focus on local details, may be more costly in late adulthood, particularly when multiple distractors are present on the display. We believe that distractor interference might be related, at least in part, to the interference of global information carried by distractors. Previous findings already suggested that global information automatically captures attention and disrupts local information processing during a visual search task (Rauschenberger & Yantis, 2001). Further experiment with manipulations of global and local interferences situations during visual search may provide an answer to this hypothesis. Moreover, even if the stimuli used in the present experiment allows for better understanding of how aging impacts global, intermediate and local levels processes, future studies will be necessary to investigate this phenomenon, for instance, the impact of the variations of visual angle or density, that are known to affect performances during global/local task (e.g., Kimchi, 1992). It will also be interesting to explore how a cueing procedure (i.e., indicating the specific location where the target could appear in the display) could affect the effortfulness of global, local and intermediate processes. The impact of such variations should be addressed in future work in order to further understand the role of the intermediate information of hierarchical stimuli during visual search.

Overall, our findings from the older group may have implications for daily life situations. As mentioned by Wynn et al. (2020), “*How older adults remember the world depends on how they see it*”, a better understanding in the key role of how age related memory decline impacts visual exploration, shows how older adults process the visual world differently than younger adults. In particular, older adults seem more likely to be impaired by a sudden salient stimulus, even if they are explicitly instructed to ignore it. Considering that older adults were more distracted by global information during local detail processing, the present findings argue that memory performance would be particularly altered when older adults have to memorize local details among several distractors. The present results may also have implications for daily life situations, such as driving. Karthaus et al. (2020) showed that during a driving simulation experiment, RTs were slower for older drivers than for younger drivers, especially when visual distractors had to be ignored. An increased difficulty with older age to focus on local details, supports the view that during a heavy traffic situation, older adults might be less efficient focusing on relevant and essential information in the environment. Finally, though recent work evidenced that performance on Navon’s hierarchical stimuli relates systematically to the ability to recognize common real objects (Gerlach & Poirel, 2018), further studies will be necessary to investigate the key role of multiple levels of processing (i.e., global, intermediate, and local) during natural stimuli and scene perception in older adults. Nevertheless, the present results provide a first clue regarding how multiple levels are processed and evolve across adulthood.

In conclusion, the current study showed that responses to visual objective stimuli are impacted by healthy aging. Our study aimed for the first time to investigate the age-related changes during global/local processing by using 1) a visual search task and 2) three-level hierarchical stimuli. The present results provided promising clues for future investigation regarding the evolution of global/local processes during healthy aging. For instance, further research could focus on the comparison of attentional cost from the to-be-ignored level among the hierarchical structure and the to-be-ignored distractor among the whole visual scene. Although healthy older participants continue to show the traditional global precedence phenomenon, they were disproportionately less efficient during detailed information processing, particularly when multiple visual information were present in the display. Taken together, the current behavioral data suggests that healthy older adults could, in some circumstances, “miss the leaves for the trees and the forest”; which should be taken into account during daily life situations.
